# A novel technique for NO.253 lymph node dissection and left colic artery preservation to avoid potential postoperative internal hernia in laparoscopic radical resection for rectal cancer

**DOI:** 10.1186/s12893-024-02492-2

**Published:** 2024-07-04

**Authors:** Wenjun Luo, Fugen Li, Chuan Qian, Tingting Lu, Yanling Xiao, Zhengwen Xu, Yingdong Jia

**Affiliations:** Department of Gastrointestinal Surgery, Suining Central Hospital, Suining, Sichuan China

**Keywords:** Preservation of left colic artery, Internal hernia, NO.253 lymph node dissection, Rectal cancer

## Abstract

**Background:**

The preservation of the left colic artery (LCA) has emerged as a preferred approach in laparoscopic radical resection for rectal cancer. However, preserving the LCA while simultaneously dissecting the NO.253 lymph node can create a mesenteric defect between the inferior mesenteric artery (IMA), the LCA, and the inferior mesenteric vein (IMV). This defect could act as a potential “hernia ring,” increasing the risk of developing an internal hernia after surgery. The objective of this study was to introduce a novel technique designed to mitigate the risk of internal hernia by filling mesenteric defects with autologous tissue.

**Methods:**

This new technique was performed on eighteen patients with rectal cancer between January 2022 and June 2022. First of all, dissected the lymphatic fatty tissue on the main trunk of IMA from its origin until the LCA and sigmoid artery (SA) or superior rectal artery (SRA) were exposed and then NO.253 lymph node was dissected between the IMA, LCA and IMV. Next, the SRA or SRA and IMV were sequentially ligated and cut off at an appropriate location away from the “hernia ring” to preserve the connective tissue between the “hernia ring” and retroperitoneum. Finally, after mobilization of distal sigmoid, on the lateral side of IMV, the descending colon was mobilized cephalad. Patients’preoperative baseline characteristics and intraoperative, postoperative complications were examined.

**Results:**

All patients’ potential “hernia rings” were closed successfully with our new technique. The median operative time was 195 min, and the median intraoperative blood loss was 55 ml (interquartile range 30–90). The total harvested lymph nodes was 13.0(range12−19). The median times to first flatus and liquid diet intake were both 3.0 days. The median number of postoperative hospital days was 8.0 days. One patient had an injury to marginal arterial arch, and after mobolization of splenic region, tension-free anastomosis was achieved. No other severe postoperative complications such as abdominal infection, anastomotic leakage, or bleeding were observed.

**Conclusions:**

This technique is both safe and effective for filling the mesenteric defect, potentially reducing the risk of internal hernia following laparoscopic NO.253 lymph node dissection and preservation of the left colic artery in rectal cancer surgeries.

**Supplementary Information:**

The online version contains supplementary material available at 10.1186/s12893-024-02492-2.

## Background

Internal hernia (IH) following laparoscopic colorectal surgery is a significant yet often under-reported complication, with prevalence ranging from 0.38 to 21% depending on the type of surgery and patient population [1–4]. Most IH cases are asymptomatic but can lead to severe complications like acute small bowel obstruction, particularly in the early postoperative months. The primary cause is often unclosed mesenteric defects. Studies have shown that routine closure of mesenteric defects significantly reduces the incidence of IH, underscoring the importance of meticulous surgical techniques to ensure safer postoperative outcomes [5–8].

Rectal cancer is one of the most prevalent types of cancer globally [9]. Surgical resection remains the only curative option. For comprehensive lymph node dissection of NO.253, the inferior mesenteric artery (IMA) is often ligated at its origin, a procedure known as high-tie [10]. Conversely, low-tie involves the preservation of the left colic artery (LCA) and the ligation of either the sigmoid artery (SA) or the superior rectal artery (SRA) below the LCA. Compared to high-tie, low-tie maintains a more sufficient blood supply to the proximal colon and does not compromise oncological outcomes, such as recurrence or survival [11, 12]. Therefore, low-tie has become the favored approach for laparoscopic radical resection for rectal cancer, where NO.253 lymphadenectomy and preservation of the LCA are carried out concurrently [13].

**Fig. 1:**
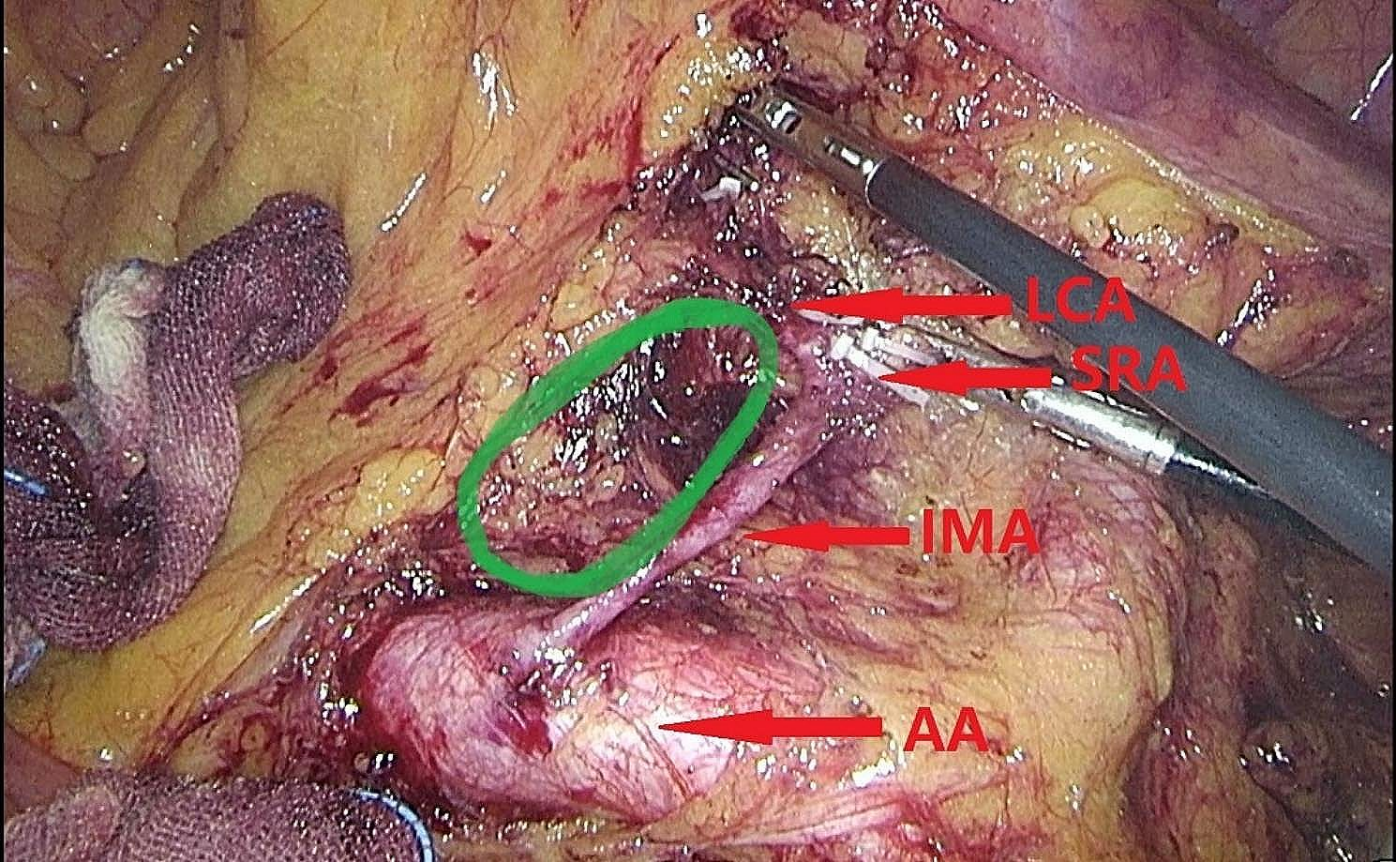
Green circle represented the mesenteric defect after NO.253 lymphadenectomy and preservation of LCA which may also become a potential hernia ring. LCA left colic artery, SRA superior rectal artery, IMA inferior mesentery artery, AA abdominal aorta

However, after NO.253 lymph node dissection, low-tie creates a mesenteric defect between the inferior mesenteric artery (IMA), left colic artery (LCA), and inferior mesenteric vein (IMV). These vessels form a foramen, which may also become a “potential hernia ring” (PHR) (Fig. 1). Furthermore, it is crucial to note that this specific defect cannot be effectively closed using techniques such as suturing surrounding tissue or employing artificial materials, unlike other types of defects. Consequently, the defect persists even after the surgery. While this specific type of internal hernia has not been previously documented, it is essential to recognize that the risk of developing an internal hernia following gastrointestinal surgery has been underestimated, and this particular defect should not be overlooked for its potential to contribute to internal hernia formation [14].

We implemented a novel technique for filling the mesenteric defect created during laparoscopic NO.253 lymphadenectomy and left colic artery (LCA) preservation. This technique involves preserving the connective tissue between the potential hernia ring (PHR) and the retroperitoneum, with the aim of reducing the risk of postoperative internal hernia. In this study, we outlined the surgical procedure and conducted a retrospective analysis to evaluate the safety and efficacy of this technique.

## Methods

### Patients

Between January 2022 and June 2022, patients with rectal cancer who underwent laparoscopic anterior resection for rectal cancer at the Department of Gastrointestinal Surgery in Suining Central Hospital were included. The indications of the novel technique were as following: (1) Patients diagnosed with rectal cancer; (2) Proposed laparoscopic anterior resection of rectal cancer with preservation of the LCA; (3)R0 resection was completed. Patients who were unable to preserve the LCA, had distant metastases, or couldn’t be radically resected were excluded. The perioperative and pathologic outcomes were prospectively collected. Postoperative clinical staging of the tumor was assessed based on the American Joint Committee on Cancer TNM classification, seventh edition. Informed consent was obtained from all patients prior to surgery. This study was approved by the ethics committee of Suining Central Hospital.

### Patient positioning and Port Placement

After the administration of general anesthesia and intubation, the patient was placed in a modifed lithotomy, 30° Trendelenburg position, and five ports were placed according to traditional practices for laparoscopic anterior resection at our department. The main surgeon was at the right side of the patient while the first assistant was at the left. The camera operator stood in front of the patient’s head. The screen was located between the two legs of the patient.

### Surgical technique

#### Initial maneuver

**Fig. 2:**
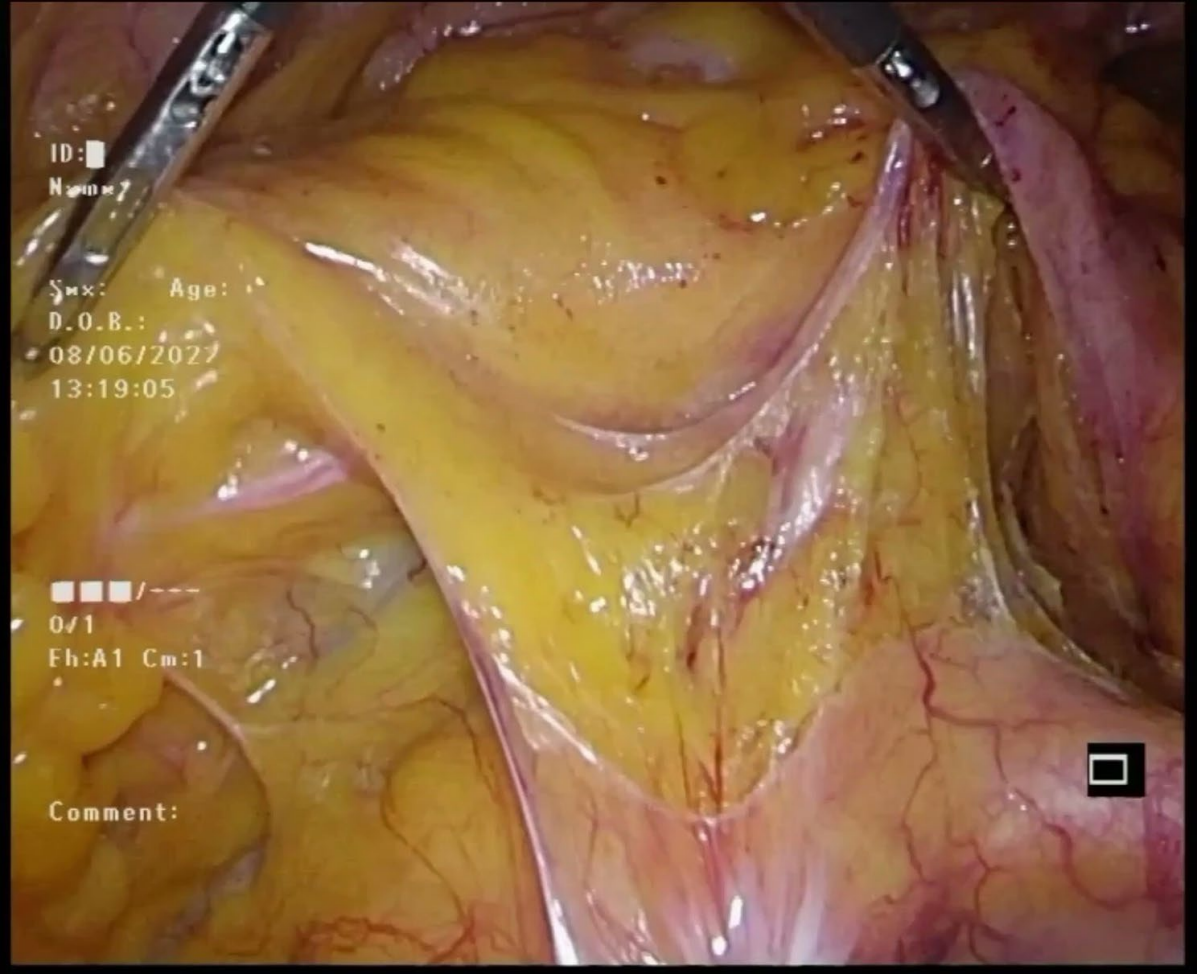
The descending colonic mesentery was pull outward and upward, while IMA was lifted upward and caudally

**Fig. 3:**
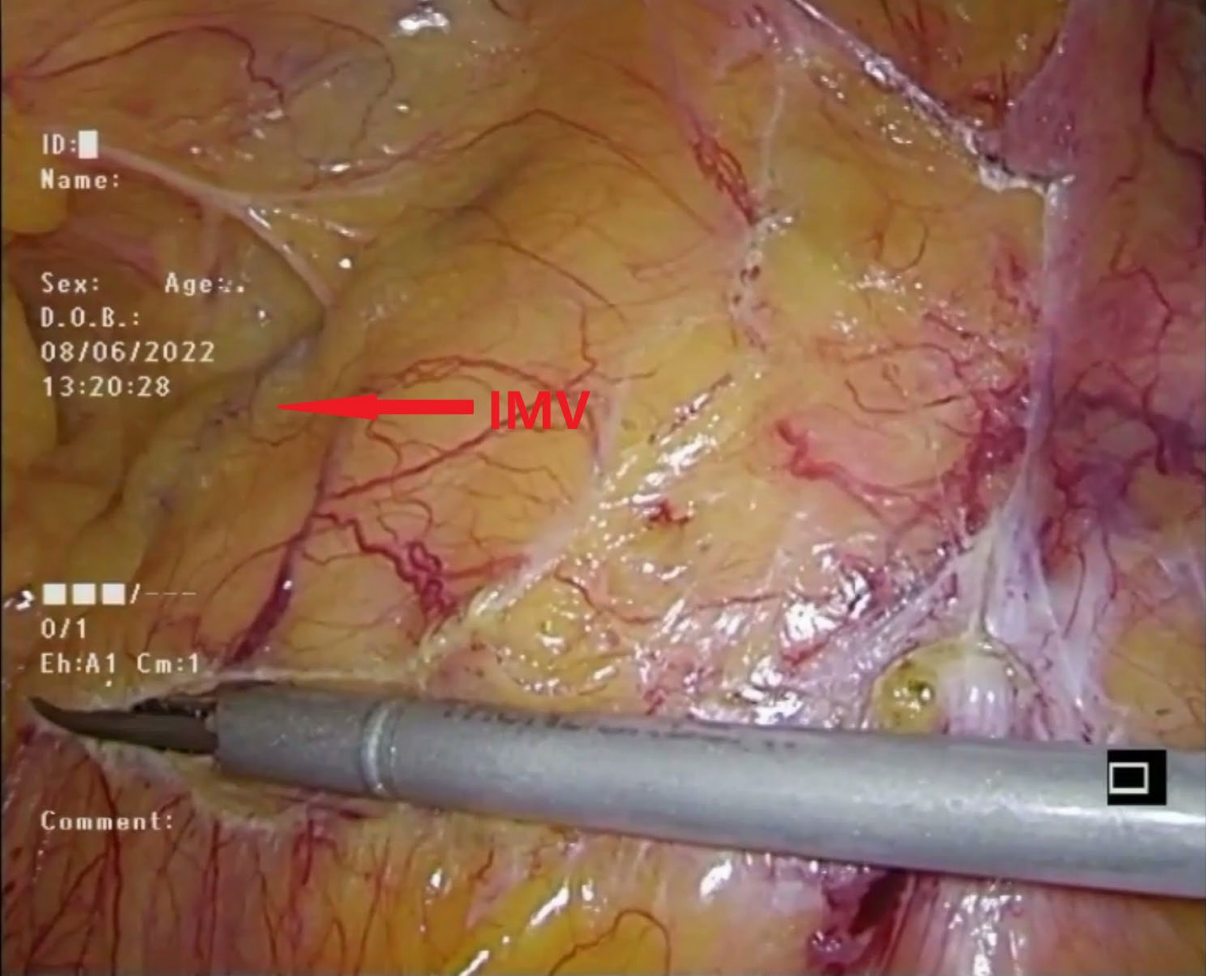
Dissected the lymph nodes at the root of the IMA (NO.253), starting from the right side, then cephalad, and along the inferior border of the duodenum to the left wall of the IMV. IMV inferior mesentery vein

To begin the procedure, the assistant performed a maneuver involving pulling the descending colonic mesentery outward and upward with one hand, while simultaneously lifting the inferior mesenteric artery (IMA) upward and caudally with the other hand (Fig. 2). Concurrently, the primary surgeon started the dissection of lymph nodes around the root of the IMA (NO.253), initiating from the right side and then progressing cephalad along the inferior border of the duodenum towards the left wall of the inferior mesenteric vein (IMV) (Fig. 3).

#### Continued dissection

**Fig. 4:**
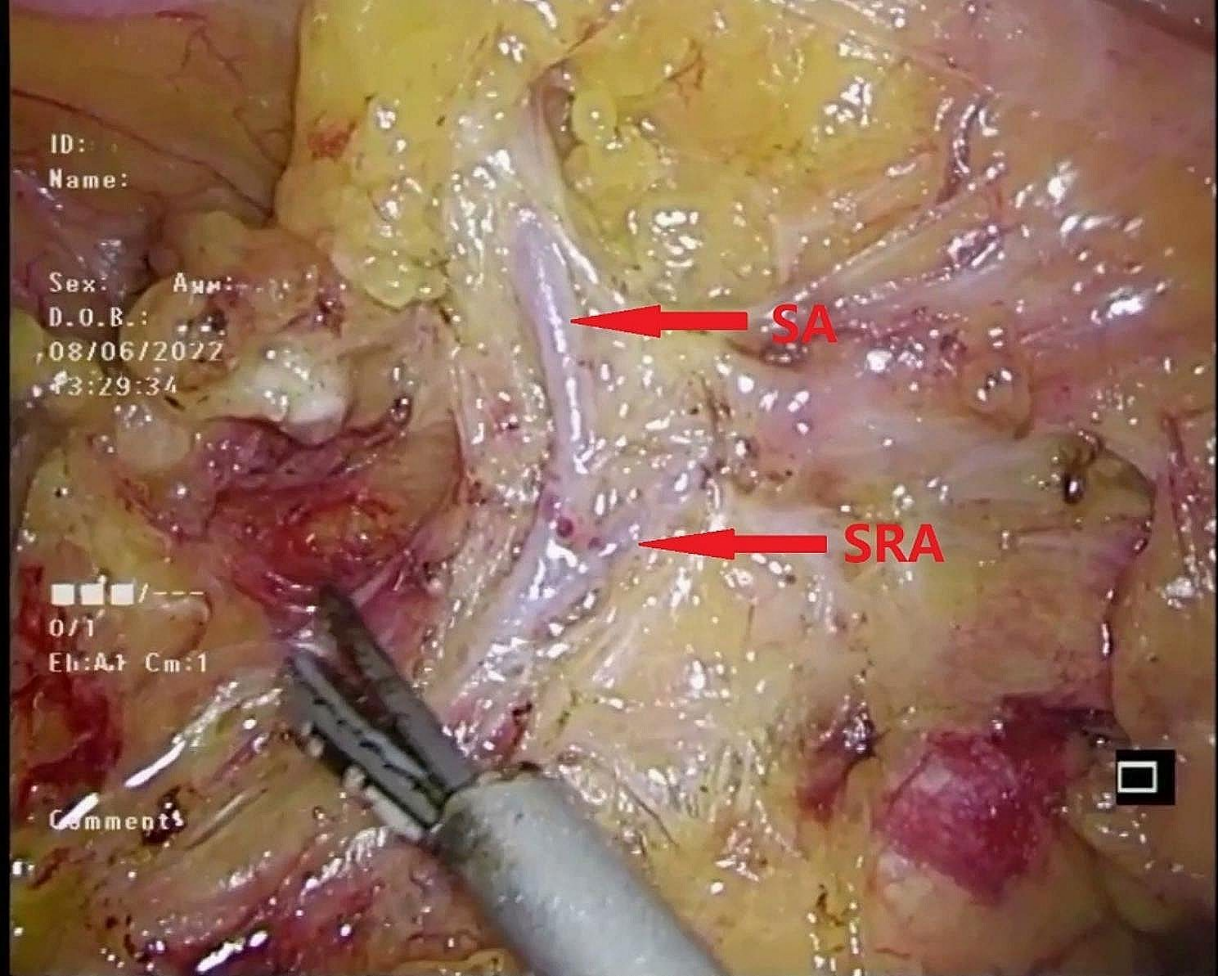
Continued to dissect the lymphatic fatty tissue on the main trunk of the IMA from its root until the LCA and SA or SRA were exposed. SA sigmoid artery, SRA superior rectal artery

The dissection then continued along the main trunk of the IMA, clearing lymphatic fatty tissue until the left colic artery (LCA) and either the sigmoid artery (SA) or the superior rectal artery (SRA) were fully exposed. This exposure of these arterial branches was crucial for facilitating the subsequent trimming of the sigmoid mesentery (Fig. 4).

#### Extending the dissection

Finally, the dissection extended outward and cephalad along the LCA to the left side wall of the IMV, returning to the point where it was initially exposed.

#### NO.253 lymph node dissection

**Fig. 5:**
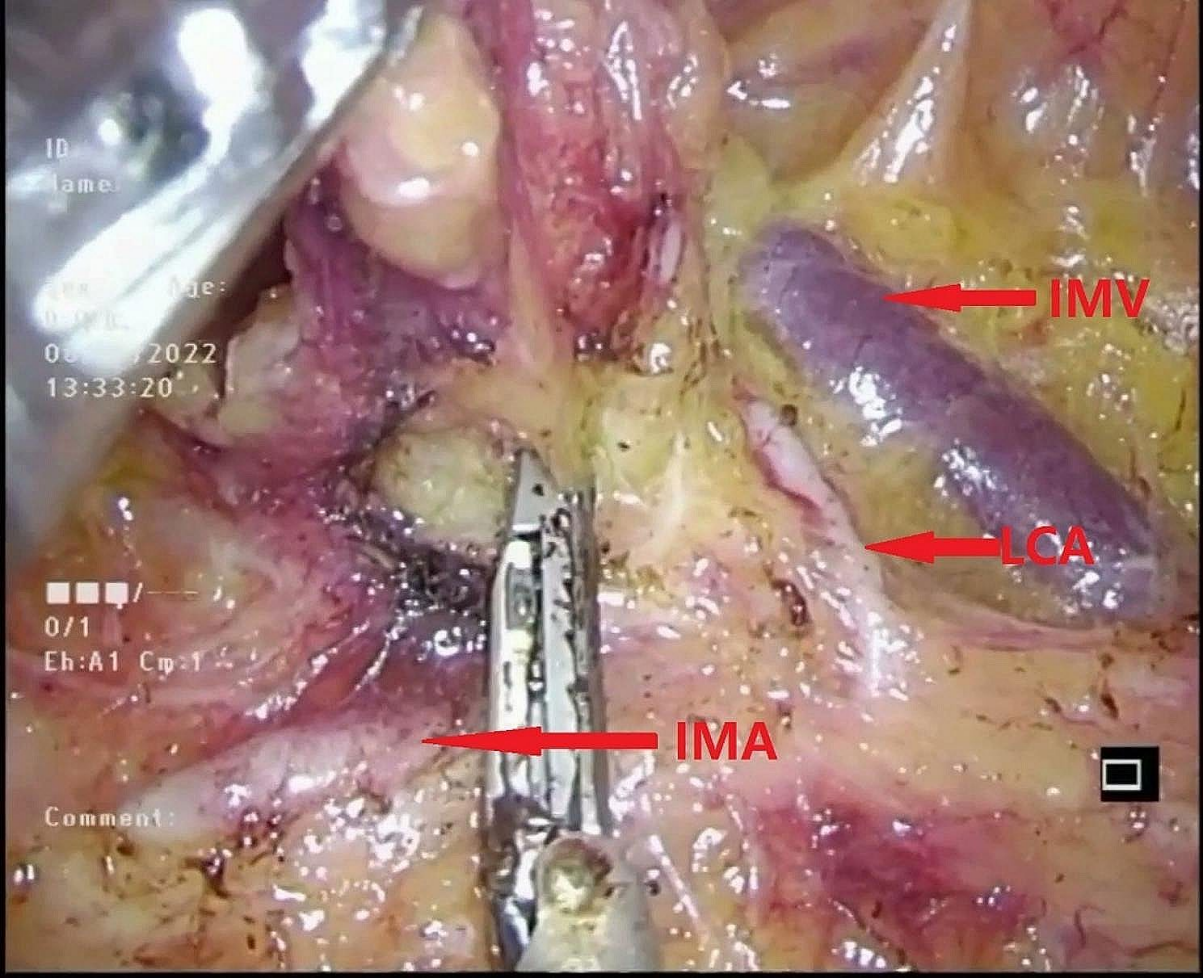
Dissected the NO.253 lymph node between the IMA, LCA and IMV

**Fig. 6:**
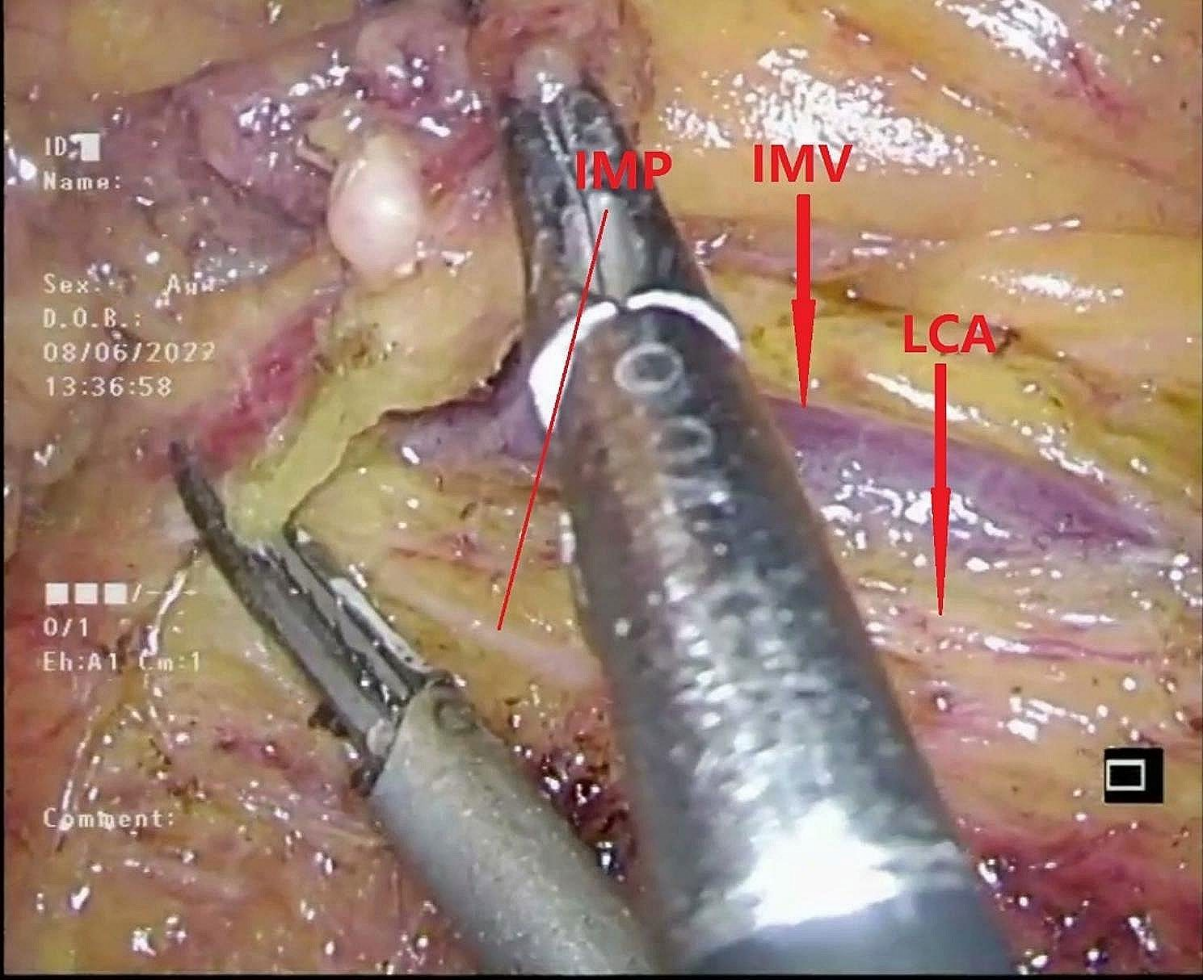
Avoid injury to the inferior mesenteric plexus. IMP: inferior mesenteric plexus

Subsequently, the NO.253 lymph node was dissected between the inferior mesenteric artery (IMA), left colic artery (LCA), and inferior mesenteric vein (IMV) (Fig. 5). When separating the space behind the mesentery, care was taken not to dissect too deeply to avoid damaging the inferior mesenteric plexus [15] (Fig. 6). Skeletalization of the IMV was deemed unnecessary due to the extremely low rate of lymph node metastasis around the IMV [16].

#### Ligating and cutting arteries

**Fig. 7:**
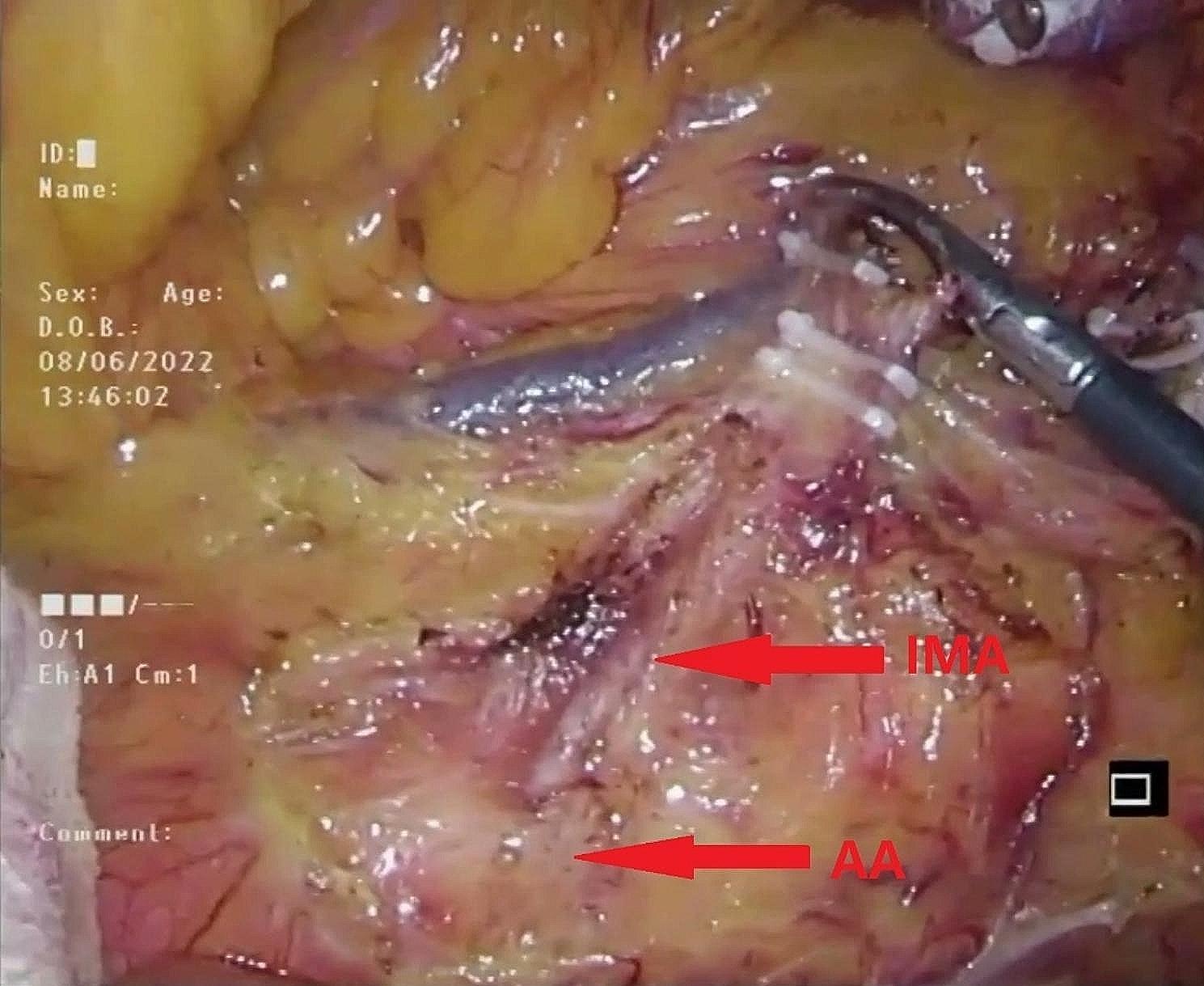
Completed the dissection of NO.253 lymph node. IMA inferior mesentery artery, AA abdominal aorta

**Fig. 8:**
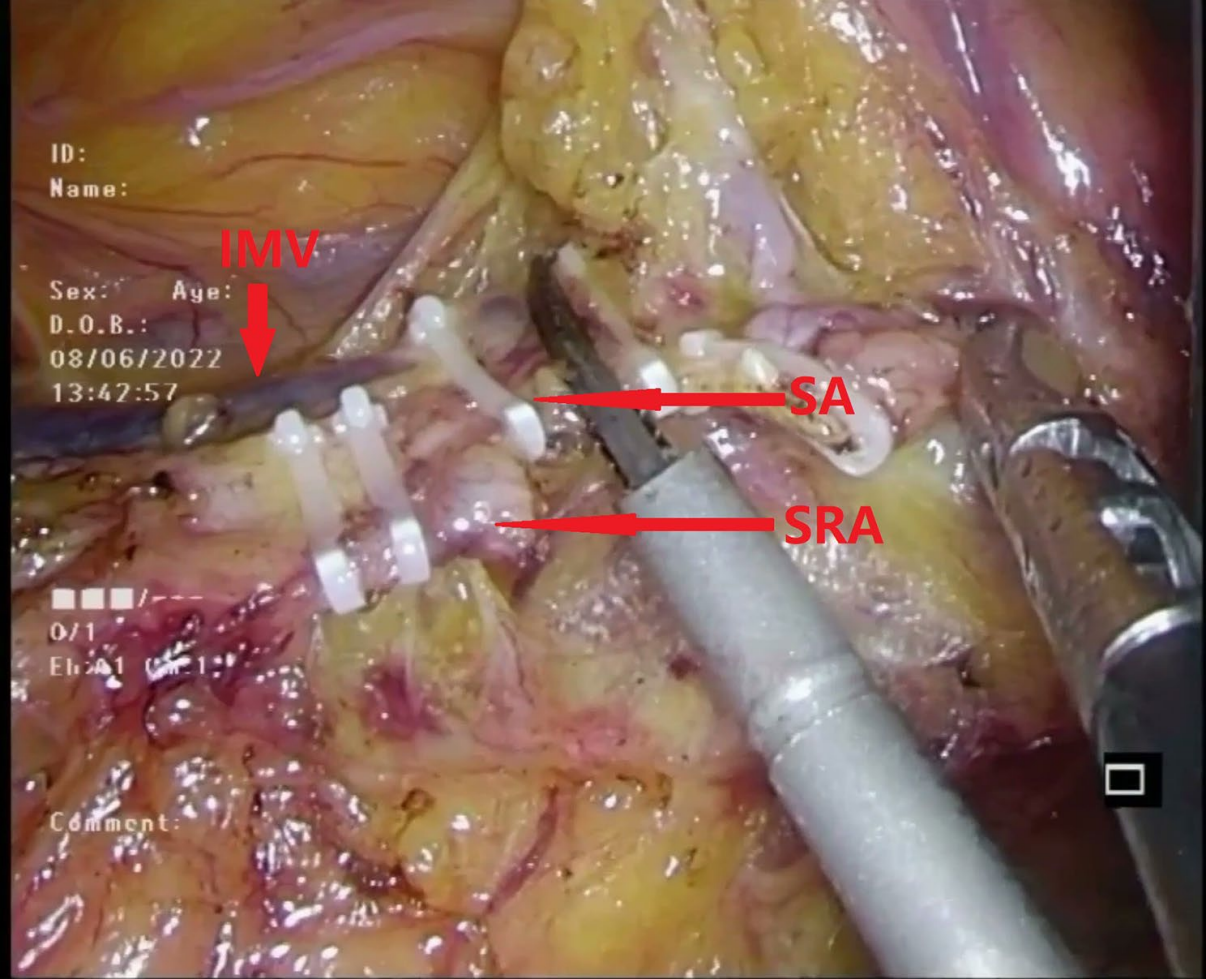
Ligated and cut off the SA or SRA and IMV at an appropriate location away from the potential “hernia ring” to preserve the connective tissue between the “potential hernia ring” and retroperitoneum

Following the dissection of the NO.253 lymph node (Fig. 7), the sigmoid artery (SA) or superior rectal artery (SRA) and the IMV were sequentially ligated and cut at a location sufficiently distant from the potential “hernia ring” to preserve the connective tissue between the PHR and retroperitoneum (Fig. 8). If the space created by the lymph node dissection exceeded the plane of the LCA, the location of the vascular ligation was adjusted caudally to better preserve the connective tissue near the PHR.

#### Bowel mobilization

**Fig. 9:**
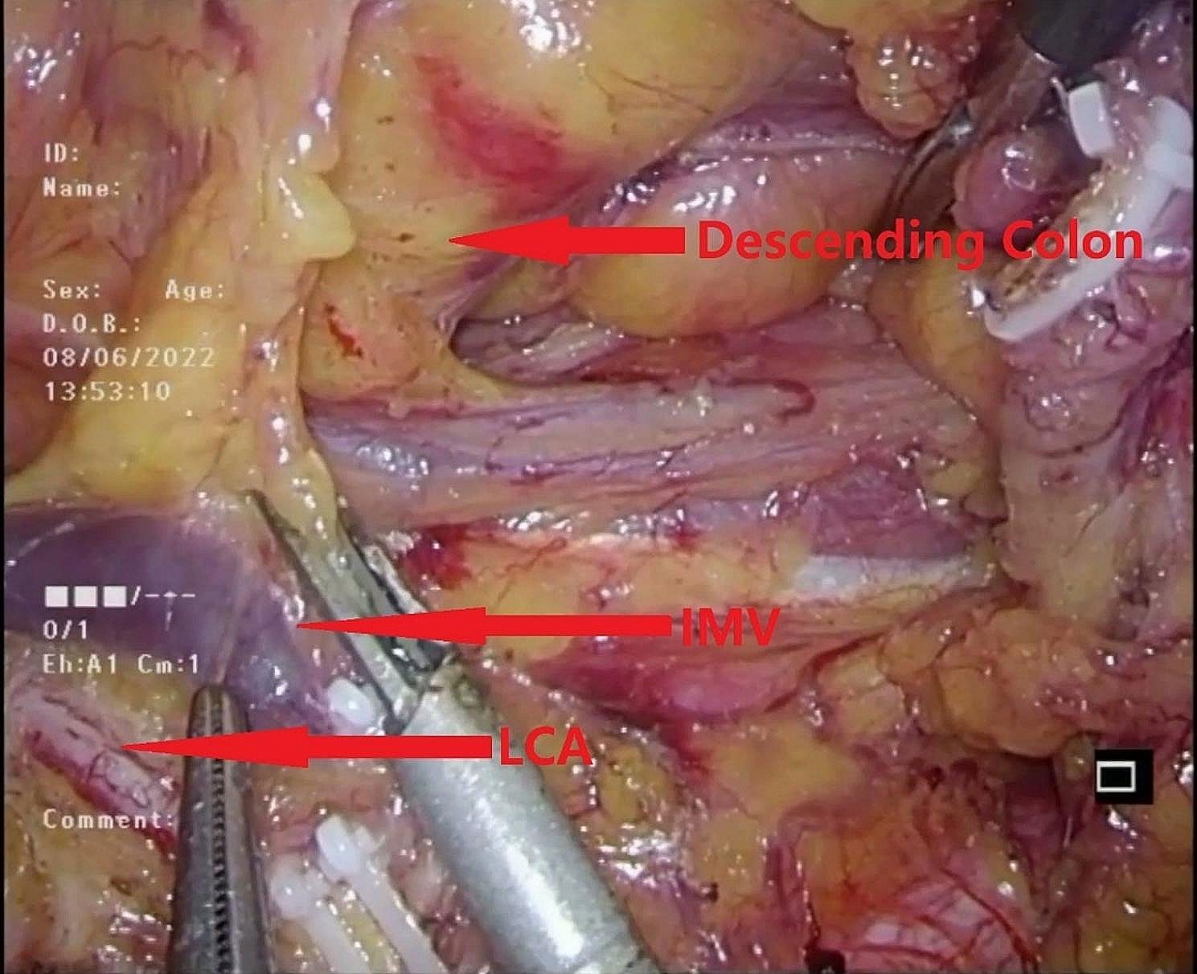
Mobilized the descending colon cephalad on the lateral side of IMV

Next, after the distal sigmoid colon was mobilized, the assistant lifted the sigmoid colon caudally and upward with one hand and the descending colon cephalad and upward with the other hand. Meanwhile, on the lateral side of the IMV, the main surgeon mobilized the descending colon cephalad (Fig. 9).

#### Preserving the connective tissue

**Fig. 10:**
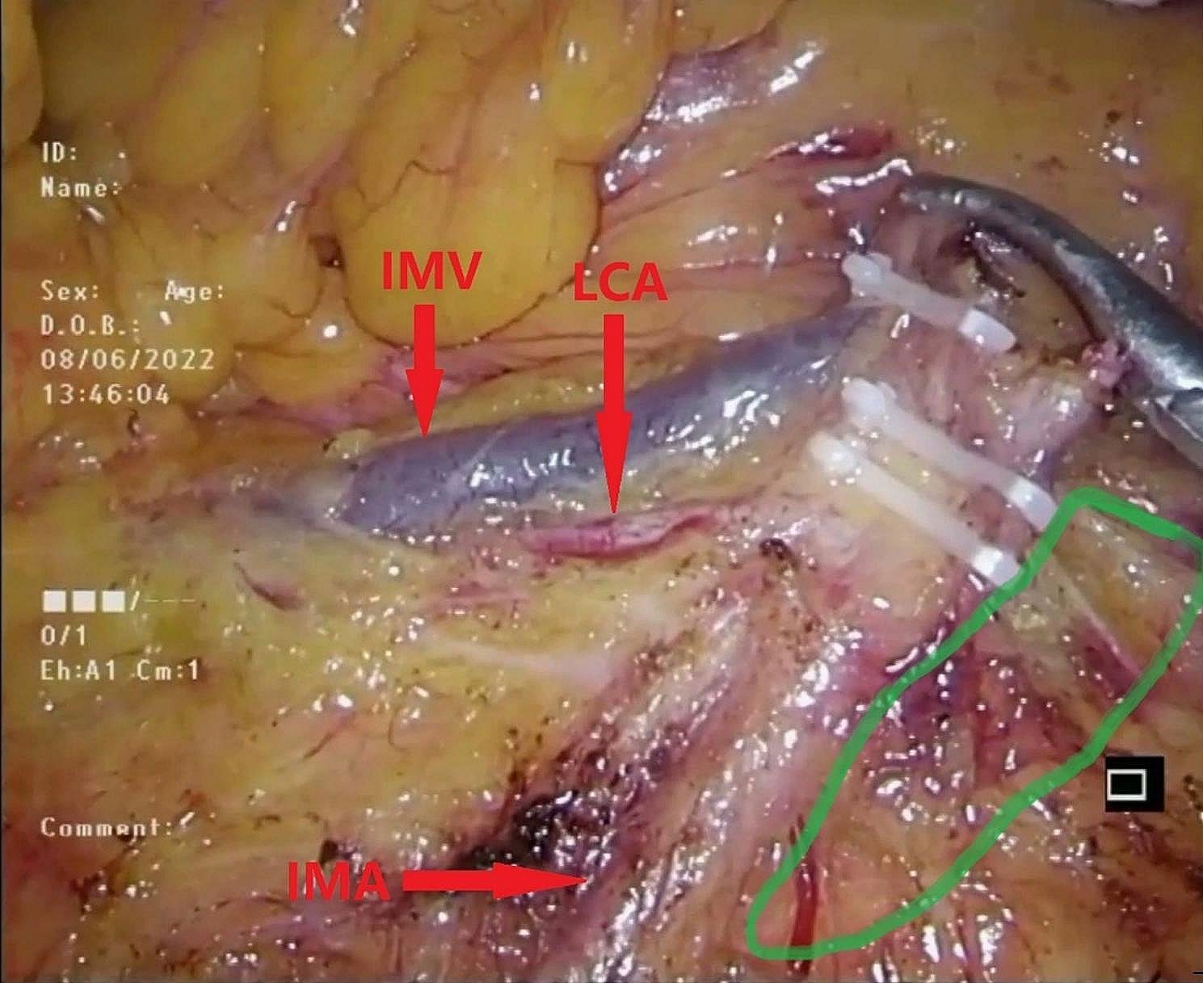
Lifted the “potential hernia ring” to check whether the meseteric defect was completely closed by the connective tissue. Blue circle represented the connective tissue between the potential “hernia ring” and retroperitoneum

Once the bowel mobilization was completed, the PHR was lifted to ensure that the connective tissue between the PHR and the retroperitoneum was completely preserved and to verify its effectiveness in preventing the incarceration of the small bowel (Fig. 10).

The surgical procedure is prescribed and presented in the supplementary video.

## Results

A total of eighteen patients who underwent laparoscopic curative anterior resection for rectal cancer were enrolled in the study between January 2022 and June 2022 in our department. Table 1 summarizes baseline patient characteristics.

**Table 1:** Baseline clinical and pathological characteristics

	No. patient (*n* = 18)
Age (y)^a^	62 (49–76)
Gender	
Men	10 (55.6%)
Women	8 (44.4%)
BMI (kg/m2)^a^	21.5 (18.5–26.8)
ASA	
I	1 (5.6%)
II	15 (83.3%)
III	2 (11.1%)
Differentiation types	
Well differentiated	3 (16.7%)
Moderately differentiated	10 (55.6%)
Poorly differentiation	5 (27.8%)
Pathological T stage	
T2	2 (11.1%)
T3	12 (66.7%)
T4	4 (22.2%)
Pathological N stage	
N0	0
N1	12 (66.7%)
N2	6 (33.3%)
Pathological M stage	
M0	18 (100%)
M1	0
Total harvested lymph nodes^a^	13.0(12–19)
Nerve invasion	3 (16.7%)

a Data are expressed as a median (range)

There were ten men and eight women included in our study with an average age of 62 (range 49–76) years and BMI of 21.5(range 18.5–26.8) kg/m^2^. There were 2, 12, and 4 patients diagnosed as T2, T3 and T4, and 12, and 6 patients diagnosed as N1, and N2, respectively. There were 3, 10 cases diagnosed as high and moderately diferentiated adenocarcinoma, and five case diagnosed as poorly diferentiated adenocarcinoma. The median number of lymph nodes retrieved was13.0. (Table 1).

All surgical procedures were performed by the same experienced laparoscopic surgeons. This technique was successfully completed in all the 18 patients. Intraoperative and postoperative characteristics are summarized in Table 2. The median operative time was 195 min, and the median intraoperative blood loss was 55 ml (interquartile range 30–90). The median times to first flatus and liquid diet intake were 3.0 days and 2 days, respectively. The median number of postoperative hospital days was 8.0. One patient had an injury to marginal arterial arch, and after mobolization of splenic region, tension-free anastomosis was achieved. No other severe postoperative complications such as abdominal infection, anastomotic leakage, or bleeding were observed. There was no 30-day re-hospitalization and mortality (Table 2).

**Table 2:** Intraoperative and postoperative outcomes

	No. patients (*n* = 18)
Operative time (min)a	195(160–225)
Intraoperative blood loss (ml)a	55(30–90)
Intraoperative complications	1
Protective stoma	2
Conversion to open surgery	0
Times to first flatus (days)a	3(2–5)
Times to liquid diet intake (days)a	2(1–5)
Postoperative hospital stay (day)a	8(7–14)
Major complications	0
Anastomotic leakage/bleeding	0/0
Mortality	0

a Data are expressed as a median (range)

Next, patients underwent various follow-up examinations according to the standard clinical schedule after surgery. To date, there have been no occurrences of internal hernias in any of the patients.

## Discussion

The low-tie technique enhances blood supply to the proximal colon without compromising long-term survival outcomes. However, the risk of internal hernia associated with this technique is often overlooked. Our innovative approach mitigates this risk by preserving connective tissue to fill the hernia ring, enhancing the safety of the low-tie technique during laparoscopic NO.253 lymphadenectomy. This method maintains standard NO.253 lymph node dissection without significantly increasing procedural complexity [17]. This is the first recognition of potential internal herniation in this context, offering a practical prevention method and highlighting the need for vigilance.

In the laparoscopic era, the pattern and outcomes of postoperative small bowel obstruction have evolved. Adhesions are significantly less common after laparoscopic surgery compared to open surgery, though not completely eliminated. However, the likelihood of postoperative hernias, including internal hernias, is higher in laparoscopic colorectal surgery [18, 19]. Incisional hernias are also more frequent in single-port laparoscopic gynecological surgery [20]. The risk of internal hernias following gastrointestinal surgery is an increasing concern among surgeons [21]. Although studies have shown that the closure of the intermesenteric space should be reconsidered during laparoscopic anterior resection, defects created during laparoscopic procedures are often left unclosed due to the additional time required for suturing [2, 3]. When internal hernias occur post-surgery, they can cause serious complications due to diagnostic challenges [22].

Recently, suturing the defect following gastrointestinal surgery has increasingly become a standard practice due to its effectiveness in reducing postoperative complications, despite the additional time required for the procedure [3, 23]. However, the risk associated with mesenteric defects, as highlighted in this study, has not been widely recognized until now. This oversight may be due to the relatively few cases that involve preservation of the left colic artery (LCA). Alternatively, while the risk might have been identified, repairing the defect with autologous tissue, similar to certain internal hernias, may not be feasible due to the lack of available surrounding mesentery or fascia [24, 25]. Moreover, the use of artificial materials, akin to repairs seen in esophageal hiatal hernias, is impractical in this context because the defect is surrounded by blood vessels, which cannot be used to anchor the mesh securely [26].

In our study, we introduced a technique that preserves connective tissue to close the defect without additional sutures, thus avoiding longer operation times. Our method simplifies the procedure by adjusting the dissection sequence and vascular ligation positions, without increasing complexity. The key is preserving the connective tissue between the pelvic hypogastric plexus (PHR) and the retroperitoneum. We dissected the NO.253 lymph node and expanded the retrosigmoid space after dividing vessels at a suitable distance from the PHR, unlike previous methods that did not preserve this tissue. This technique closely resembles previous procedures but requires caution to avoid excessive expansion near the PHR before dividing vessels [27].

When employing this surgical technique, several key technical considerations are crucial for optimal outcomes and minimizing complications. First, if lymph nodes encircle the root of the inferior mesenteric artery (IMA), the left colic artery (LCA) should not be preserved; instead, ligation at the IMA root is necessary. Second, significantly enlarged NO.253 lymph nodes should be clipped at their root to prevent postoperative lymphatic leakage [28]. Third, when dissecting the NO.253 lymph node, it is important to protect the inferior mesenteric plexus to help preserve genitourinary function post-surgery [29]. Therefore, careful upward lifting of the NO.253 lymph node and the use of blunt dissection techniques are crucial. Fourth, assess the jejunum’s location before lymph node dissection to avoid damage. Lastly, retain the LCA only if it ensures a tension-free anastomosis, considering the sigmoid colon length and tumor size.

To ensure successful intraoperative vascular dissection, familiarity with variations of the sigmoid artery is crucial, or alternatively, using CT angiography may help. Care should be taken to prevent damage to the marginal artery when trimming the mesentery [30]. In our study, one patient had a marginal arterial arch injury, resolved by mobilizing the splenic region for a tension-free anastomosis. This step isn’t routine in anterior resection, especially with longer sigmoid colons in Chinese patients. Assess the marginal arterial arch position carefully if the mesentery is short or thick [31]. During trimming, the assistant should pull the mesentery outward with one hand while guiding the inferior mesenteric artery (IMA) caudally with the other, avoiding pulling caudally with both hands to prevent injury to the marginal vessels.

The present study shows promise but has significant limitations. The small sample size may limit the generalizability of the findings, making it difficult to apply the results to a broader population. Additionally, the short follow-up period restricts the ability to observe long-term outcomes and complications. The single-center design further limits the scope, as it may not reflect variations across different institutions. These limitations suggest that larger, multi-center studies with extended follow-up periods are necessary to comprehensively evaluate the technique’s effectiveness and safety, thereby improving the generalizability of the results.

In summary, while the potential risk of internal hernia following laparoscopic radical resection for rectal cancer with LCA preservation and NO.253 lymph node dissection remains an area of concern, our innovative technique aims to address this issue. By preserving the connective tissue between the potential hernia ring (PHR) and the retroperitoneum, we propose a method that could potentially reduce the risk of postoperative internal hernia. However, further validation of this technique’s safety and efficacy is essential, and should be pursued through a large-scale, multi-center trial in the future.

### Electronic supplementary material

Below is the link to the electronic supplementary material.

Supplementary Material 1

## Data Availability

The datasets used and analyzed during the current study are available from the corresponding author on reasonable request.
